# The Pathologic Complete Response Ratio of Liver Metastases Represents a Valuable Prognostic Indicator

**DOI:** 10.3389/pore.2022.1610663

**Published:** 2022-09-06

**Authors:** Yanbo Xu, Jiarui He, Weihao Li, Weili Zhang, Songran Liu, Jiahua He, Zhizhong Pan, Zhenhai Lu, Jianhong Peng, Junzhong Lin

**Affiliations:** ^1^ Department of Colorectal Surgery, Sun Yat-sen University Cancer Center, State Key Laboratory of Oncology in South China, Collaborative Innovation Center for Cancer Medicine, Guangzhou, China; ^2^ Zhongshan School of Medicine, Sun Yat-sen University, Guangzhou, China; ^3^ Department of Pathology, Sun Yat-sen University Cancer Center, State Key Laboratory of Oncology in South China, Collaborative Innovation Center for Cancer Medicine, Guangzhou, China

**Keywords:** recurrence, colorectal cancer, prognosis, liver metastasis, pathologic complete response ratio

## Abstract

**Background and Objectives:** The aim of this study was to evaluate the role of the pathologic complete response ratio of liver metastases (PCRRLM) in predicting the prognosis and recurrence of colorectal cancer liver metastases (CRLM).

**Methods:** A total of 305 CRLM patients who underwent preoperative chemotherapy followed by hepatectomy were included. PCRRLM was defined as the number of liver metastases exhibiting pathologic complete response (PCR) divided by the number of total resected liver metastases. The Kaplan–Meier method was used to calculate survival, and differences were examined by the log-rank test. Univariate and multivariate analyses were performed to identify the predictors of PCRRLM, recurrence-free survival (RFS) and overall survival (OS).

**Results:** Among the 305 included patients, 44 (14.4%) achieved a PCRRLM ≥0.50 (including PCRRLM = 1), and 261 (85.6%) achieved a PCRRLM <0.50 (including PCRRLM = 0). Patients of an older age (≥55 years old) and those with higher carcinoembryonic antigen (CEA) levels (≥5 ng/ml) were less likely to achieve a PCRRLM ≥0.50. In the multivariate analysis, PCRRLM≥ 0.50 (vs. < 0.50, HR [95% CI]: 0.67 [0.46–0.99], *p* = 0.043) was associated with better RFS. Positive lymph node status (vs. negative, HR [95% CI]: 1.46 [1.04–2.05], *p* = 0.028) and TBS ≥5 (vs. < 5, HR [95% CI]: 1.44 [1.02–2.04], *p* = 0.038) were associated with worse RFS.

**Conclusion:** PCRRLM was significantly associated with long-term RFS after preoperative chemotherapy and CRLM resection. Thus, it may be a valuable indicator of recurrence in CRLM patients.

## Introduction

Colorectal cancer (CRC) is the third most common type of cancer worldwide, with estimated 1.8 million new cases and 0.91 million deaths in 2020 [1]. Approximately 25–30% of CRC patients develop colorectal liver metastases (CRLM) during the course of the disease [2, 3]. Hepatectomy is currently the most effective and potentially curative treatment for patients with CRLM, with a 5-year survival rate of 40%–50% [4–7]. In recent years, preoperative chemotherapy has been widely used to treat CRLM, not only to downsize the lesions, ensuring their surgical resectability and the control of micrometastases [8, 9] but also to prolong progression-free survival [10]. Unfortunately, more than half of patients still develop postoperative recurrence within 2 years [11].

Many clinical factors have been used to predict the survival of patients with CRLM after hepatectomy, including the tumor size and number of lesions [8, 12–14], carcinoembryonic antigen (CEA) level [12, 15], node-positive primary disease [12, 16], clinical risk score (CRS) [17–19], and resection margin involvement [16, 20]. With the development of preoperative chemotherapy, new clinicopathological factors have emerged. Pathologic complete response (PCR), which is defined as the absence of any residual cancer cells, has been proven to confer an extremely favorable response to chemotherapy and can serve as an indicator of good prognosis [21–27]. However, its applicability to CRLM is limited because only approximately 5% of these patients achieve all-lesion PCR, with the vast majority achieving only partial-lesion PCR [23, 28]. Among the patients who achieve partial PCR, the number of resected liver metastases required to achieve PCR varies, and little research has been conducted to evaluate the relationship between the number of liver metastases exhibiting PCR and the prognosis of patients.

In this study, we applied a novel method to evaluate the pathologic response of CRLM patients. We defined the pathologic complete response ratio of liver metastases (PCRRLM) as the number of liver metastases exhibiting PCR divided by the number of total resected liver metastases. Then, we explored the value of the PCRRLM as an indicator of recurrence after preoperative chemotherapy and the need for liver resection in patients with CRLM.

## Materials and Methods

### Patient Selection

In this retrospective study, we reviewed the medical records of consecutive patients with CRLM who underwent liver resection between September 1999 and August 2020 at Sun Yat-sen University Cancer Center. A total of 305 patients who underwent curative liver resection for CRLM following preoperative chemotherapy were included in this study. The inclusion criteria were as follows: 1) radical resection of both the colorectal primary tumor and liver metastases; 2) histologically confirmed colorectal adenocarcinoma; 3) preoperative chemotherapy before hepatectomy; and 4) complete clinical and pathological information. Clinical information including patient demographics, tumor characteristics, treatment details, and follow-up data was collected from the electronic medical record system. All procedures performed in this study were in conducted accordance with the ethical standards of the World Medical Association Declaration of Helsinki and were approved by the Institutional Research Ethics Committee of Sun Yat-sen University Cancer Center (approval number: 2020-309-01). Informed consent was waived because of the observational and retrospective nature of the study, and all patient data were kept strictly confidential.

### Treatments

All included patients received preoperative chemotherapy according to the recommendations of a multidisciplinary team (MDT). XELOX (oxaliplatin and capecitabine), FOLFOX (oxaliplatin, leucovorin [LV] and 5-fluorouracil [5-FU]), FOLFIRI (irinotecan, LV and 5-FU) and capecitabine were used based on the recommended doses in the NCCN guidelines. Computerized tomography (CT) or magnetic resonance imaging (MRI) was performed every 3 months to assess the tumor response according to Response Evaluation Criteria in Solid Tumors, version 1.1 [29]. Hepatectomy was performed for resectable liver lesions after preoperative chemotherapy. Postoperative adjuvant chemotherapy was administered based on the pathological stage and tolerance of the patient. Generally, the postoperative regimens used were consistent with the preoperative regimens.

### Pathologic Response Examination

CRLM was localized by imaging before the operation. All resected tumors in each CRLM patient were sampled. The hepatectomy specimens were sectioned into 5 µm-thick slices. The hematoxylin-eosin-stained sections were carefully examined by two independent pathologists who were blinded to the patient data, treatment regimen and outcome. PCR was defined as the absence of any residual cancer cells in each tissue section, and PCRRLM was defined as the number of liver metastases exhibiting PCR divided by the number of total resected liver metastases of each patient.

### Parameter Measurements

Primary tumors were staged according to the eighth edition of the American Joint Committee of Cancer (AJCC) TNM staging system. The liver metastasis characteristics such as number, size, and distribution were assessed by enhanced abdominal nuclear MRI at the time of diagnosis. Synchronous metastases were defined as liver metastases detected at the time of diagnosis or within 6 months after radical resection of the primary tumor, while metachronous metastases were defined as liver metastases detected more than 6 months after radical resection of the primary colorectal tumor [30]. The CEA level was measured before hepatectomy, and the cutoff value was 5 ng/ml according to the normal reference interval. As previously described [31], the tumor burden score (TBS) was defined as the distance from the origin on a Cartesian plane to the coordinates (x, y) = (maximal tumor size in centimeters, number of tumors). The cutoff values for age, the number and maximum size of liver metastases, and TBS were based on the median values. CRS was calculated according to 5 independent preoperative risk factors identified in previous studies [18, 32]: 1) node positive status of primary tumor (pathological), 2) disease-free interval from the primary to discovery of the liver metastases <12 months, 3) number of metastases >1 (radiological), 4) size of the largest metastases >5 cm (radiological) and 5) preoperative CEA level >200 ng/ml, and each item represents one point. The cutoff value was 3 points, with a CRS of 0-2 points indicating a lower recurrence risk and a CRS of 3-5 points indicating a higher recurrence risk.

### Follow-Up

After liver resection, follow-up was conducted through clinical visits every 3 months for the first 2 years and then semiannually until 5 years. The evaluations included the clinical examination and assessment of CEA levels and CT imaging of the chest, abdomen, and pelvis at 3, 6, 12, and 18 months, 2 years, and annually thereafter. Liver MRI was applied to confirm suspicious lesions indicated on CT or in patients with negative CT results but increased CEA levels. The final follow-up visit was performed in June 2022. Overall survival (OS) was defined as the time interval from liver resection to the date of death due to any cause or the date of the last follow-up visit, while recurrence-free survival (RFS) was calculated as the time interval from liver resection to disease recurrence, death from disease, or last follow-up. Random censoring was applied to patients without recurrence or death at the last follow-up. According to previous studies [33], early recurrence was defined as disease recurrence or death occurring within 6 months of liver resection, and late recurrence was defined as disease recurrence or death occurring at least 6 months after liver resection.

### Statistical Analyses

Categorical variables are expressed as numbers with percentages, and continuous variables are expressed as medians with interquartile ranges. Intergroup comparisons were performed using the chi-square test. The chi-square test and Fisher’s exact test were used for categorical variables, and Student’s t test was used for continuous variables to compare baseline characteristics. Univariate and multivariate logistic regression analyses were performed to determine the predictors of recurrence and PCRRLM, and odds ratios (ORs) and 95% confidence intervals (CIs) were subsequently calculated. Survival curves were plotted using the Kaplan–Meier method, and differences between groups were assessed with the log-rank test. Univariate and multivariate analyses of Cox proportional hazards regression models were performed to evaluate the association of the relevant clinicopathological factors with prognosis. Hazard ratios (HRs) with 95% CIs were then calculated. All statistical analyses were conducted using SPSS software version 25.0 (IBM Corp., Armonk, NY, United States) and GraphPad Prism version 7.0, and *p* < 0.05 was considered statistically significant.

## Results

### Patient Characteristics

The clinicopathological characteristics of the patients are summarized in Table 1. A total of 305 patients were enrolled in this study, with a median age of 55.2 years (range 26–82 years); 213 (69.8%) were male, and 92 (30.2%) were female. The primary tumor location was the colon in 187 patients (61.3%) and the rectum in 118 patients (38.7%). The median CRLM number was 3 (range 1–14), and the median size was 3.0 cm (range 0–14 cm). Regarding first-line therapy regimens, 116 (38.0%) received FOLFIRI, 94 (30.8%) received FOLFOX, 75 (24.6%) received XELOX, and 20 (6.6%) received other regimens. The median number of chemotherapy cycles was five (range 1–16). In addition, a total of 135 patients received targeted therapy, of whom 84 (62.2%) received bevacizumab and 51 (37.8%) received cetuximab. The median CRS was 2, with 159 patients (52.1%) having a score between 0 and 2 points and 146 (47.9) having a score between 3 and 5 points. The median TBS was 4.72 points (interquartile range [IQR], 3.16–6.80, range, 1.00–15.23), with 151 patients (49.5%) scoring ≥5 points. Among the 305 patients, 231 (75.7%) received postoperative chemotherapy. During the median follow-up time of 64.87 months (range, 2.23–177.20 months), 243 patients (79.7%) experienced tumor recurrence, including 89 patients (29.2%) who experienced early recurrence (within 6 months) and 161 (52.8%) who passed away.

**TABLE 1:** Clinicopathological characteristics of the CRLM patients.

Characteristics	N (%)
Age (years, median [range])	55 (26–82)
Sex	
Female	92 (30.2)
Male	213 (69.8)
LM number (median [range])	3 (1–14)
LM diameter (cm, median [range])	3 (0–14)
CRS (median [range])	2 (0–4)
TBS (median [range])	4.72 (1–15.23)
Primary tumor location	
Colon	187 (61.3)
Rectum	118 (38.7)
Pathology	
Adenocarcinoma	276 (90.5)
Mucinous adenocarcinoma	17 (5.6)
Unknown	12 (3.9)
Lymph node metastases	
Negative	139 (45.6)
Positive	166 (54.4)
CEA (ng/ml)	
≤5	119 (39.0)
>5	186 (61.0)
Preoperative chemotherapy regimen	
Irinotecan-based	116 (38.0)
Oxaliplatin-based	169 (55.4)
Others	20 (6.6)
Preoperative targeted therapy	
Bevacizumab	84 (27.5)
Cetuximab	51 (16.7)
No	170 (55.7)
Interventional therapy	
No	226 (74.1)
Yes	79 (25.9)
Synchronous liver metastases	
No	60 (19.7)
Yes	245 (80.3)
Hepatic lobe involvement	
Double	166 (54.4)
Single	139 (45.6)
Postoperative chemotherapy	
No	74 (24.3)
Yes	231 (75.7)

Abbreviations: CEA, carcinoembryonic antigen; CRLM, colorectal liver metastases; CRS, clinical risk score; LM, liver metastases; TBS, tumor burden score.

### Pathologic Complete Response Ratio of Liver Metastases

In this study, 15 patients (4.9%) achieved a complete pathologic response (PCRRLM = 1), 29 patients (9.5%) achieved a PCRRLM ≥0.50 but <1, 44 patients (14.4%) achieved a PCRRLM <0.50 but >0, and 217 patients (71.1%) showed no sign of pathologic response (PCRRLM = 0). Considering the frequency of PCRRLM and its influence on RFS and OS, the whole cohort was divided into two groups for further analyses: the PCRRLM ≥0.50 group (*n* = 44, including PCRRLM = 1) and the PCRRLM <0.50 group (*n* = 261, including PCRRLM = 0). The baseline characteristics of the patients with a PCRRLM ≥0.50 and those with a PCRRLM <0.50 are shown in Table 2. A PCRRLM ≥0.50 was significantly associated with a younger age (<55 years old, *p* = 0.011), smaller liver metastasis diameter (≤3 cm, *p* = 0.005), lower CEA level (≤5 ng/ml, *p* < 0.001), and lower CRS (<3, *p* = 0.048) and TBS (<5, *p* = 0.027). A PCRRLM <0.50 was significantly more common in colon cancer patients (*p* = 0.020).

**TABLE 2:** Baseline characteristics of the patients with a PCRRLM ≥0.50 and <0.50.

Characteristics	PCRRLM ≥0.50 (*n* = 44)	PCRRLM <0.50 (*n* = 261)	*p* value
Age (years)			**0.011**
<55	29 (65.9)	118 (45.2)	
≥55	15 (34.1)	143 (54.8)	
Sex			0.796
Female	14 (31.8)	78 (29.9)	
Male	30 (68.2)	183 (70.1)	
LM number			0.762
<3	23 (52.3)	130 (49.8)	
≥3	21 (47.7)	131 (50.2)	
LM diameter (cm)			**0.005**
<3	30 (68.2)	118 (45.2)	
≥3	14 (31.8)	143 (54.8)	
Primary site			**0.020**
Colon	20 (45.5)	167 (64.0)	
Rectum	24 (54.5)	94 (36.0)	
Lymph node metastases			0.335
Negative	23 (52.3)	116 (44.4)	
Positive	21 (47.7)	145 (55.6)	
Synchronous liver metastases			0.276
No	6 (13.6)	54 (20.7)	
Yes	38 (86.4)	207 (79.3)	
CEA (ng/ml)			**<0.001**
≤5	32 (72.7)	87 (33.3)	
>5	12 (27.3)	174 (66.7)	
Hepatic lobe involvement			0.196
Double	20 (45.5)	146 (55.9)	
Single	24 (54.5)	115 (44.1)	
Preoperative chemotherapy regimen			0.97
Irinotecan-based	16 (36.364)	100 (38.314)	
Oxaliplatin-based	25 (56.818)	144 (55.172)	
Others	3 (6.818)	17 (6.513)	
Postoperative chemotherapy			0.902
No	11 (25.0)	63 (24.1)	
Yes	33 (75.0)	198 (75.9)	
CRS			**0.048**
<3	29 (65.9)	130 (49.8)	
≥3	15 (34.1)	131 (50.2)	
TBS			**0.027**
<5	29 (65.9)	125 (47.9)	
≥5	15 (34.1)	136 (52.1)	

Abbreviations: CEA, carcinoembryonic antigen; CRS, clinical risk score; LM, liver metastases; PCRRLM, pathologic complete response ratio of liver metastases; TBS, tumor burden score. The bold values in table indicate *p* < 0.05.

The univariable and multivariable logistic regression (Table 3) revealed that age ≥55 years old (vs. < 55 years old; OR [95% CI]: 0.47 [0.23–0.96], *p* = 0.038) and CEA level >5 ng/ml (vs. ≤ 5; OR [95% CI]: 0.23 [0.11–0.48], *p* < 0.001) were related to a lower likelihood of achieving a PCRRLM ≥0.50.

**TABLE 3:** Univariable and multivariable logistic regression analyses predicting risk factors for PCRRLM.

Variables	Univariable		Multivariable	
	OR (95% CI)	*p* value	OR (95% CI)	*p* value
Age (years): ≥ 55 vs. < 55	0.43 (0.22, 0.84)	**0.013**	0.47 (0.23, 0.96)	**0.038**
Sex: male vs. female	0.91 (0.46, 1.82)	0.796		
Primary tumor site: rectum vs. colon	2.13 (1.12, 4.07)	**0.021**	0.53 (0.27–1.06)	0.072
Lymph node metastases: positive vs. negative	0.73 (0.38, 1.39)	0.336		
Pathology: adenocarcinoma vs. mucinous adenocarcinoma	2.79 (0.36–21.63)	0.326		
CEA (ng/ml): > 5 vs. ≤ 5	0.19 (0.09, 0.38)	**<0.001**	0.23 (0.11, 0.48)	**<0.001**
Chemotherapy regimen: irinotecan-based vs. oxaliplatin-based	0.92 (0.47–1.81)	0.813		
Chemotherapy cycles: > 4 vs. ≤ 4	1.10 (0.58–2.11)	0.767		
Synchronous LM: Yes vs. No	1.65 (0.66, 4.13)	0.280		
LM number: ≥ 3 vs. < 3	0.75 (0.40, 1.43)	0.386		
LM diameter (cm): ≥ 3 vs. < 3	0.39 (0.19, 0.76)	**0.006**	0.57 (0.25–1.31)	0.183
Hepatic lobe involvement: single vs. double	1.52 (0.80, 2.90)	0.199		
CRS: ≥ 3 vs. < 3	0.51 (0.26, 1.00)	0.051		
TBS: ≥ 5 vs. < 5	0.48 (0.24, 0.93)	**0.029**	0.88 (0.38–2.06)	0.775

Abbreviations: CEA, carcinoembryonic antigen; CI, confidence interval; CRS, clinical risk score; LM, liver metastases; OR, odds ratio; PCRRLM, pathologic complete response ratio of liver metastases; TBS, tumor burden score. The bold values in table indicate *p* < 0.05.

### Survival

**FIGURE 1:**
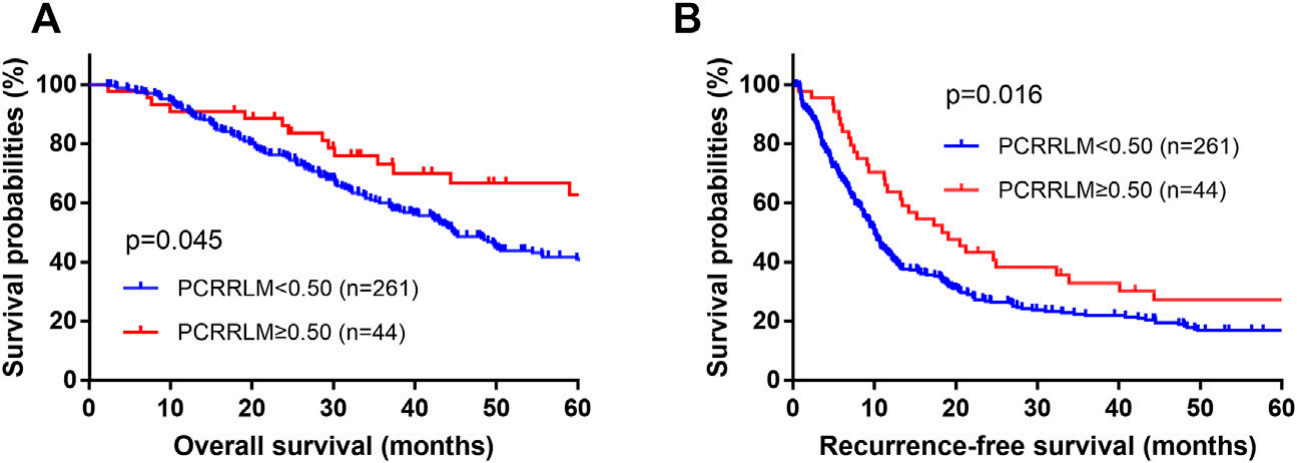
Plots of patient survival stratified by the PCRRLM. **(A)** Overall survival plots of patients stratified by the PCRRLM. **(B)** Recurrence-free survival plots of patients stratified by the PCRRLM. PCRRLM, pathologic complete response ratio of liver metastases.

The median follow-up durations for patients with a PCRRLM ≥0.50 and those with a PCRRLM <0.50 were 84.23 and 63.33 months, respectively. Both RFS and OS were significantly better in patients with a PCRRLM ≥0.50 than in those with a PCRRLM <0.50 (5-year OS rates, 62.75% vs. 41.06%, *p* = 0.045; 5-year RFS rates, 27.18% vs. 16.82%, *p* = 0.016, Figure 1).

**FIGURE 2:**
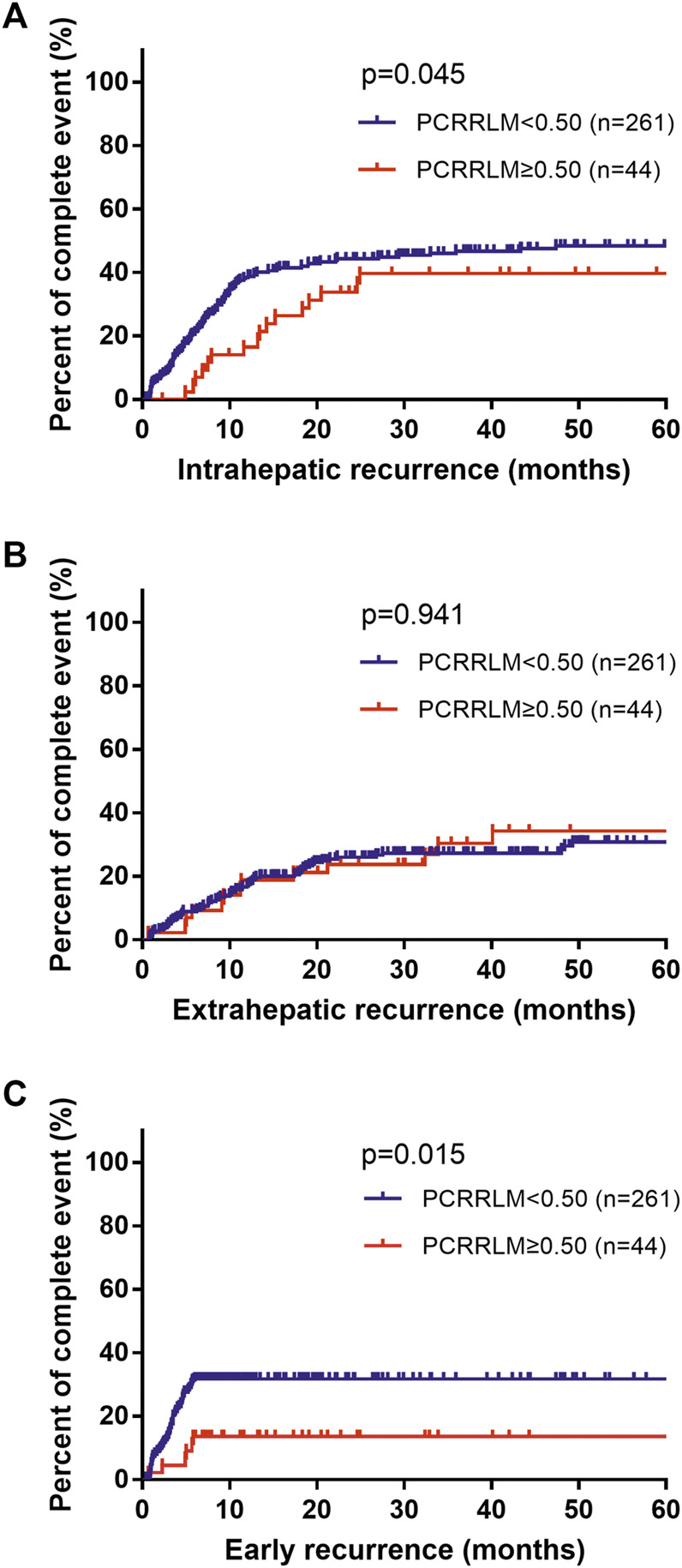
Death percent of patients with different recurrence rates stratified by the PCRRLM. **(A)** Intrahepatic recurrence from liver resection stratified by the PCRRLM. **(B)** Extrahepatic recurrence from liver resection stratified by the PCRRLM. **(C)** Early recurrence (within 6 months) from liver resection stratified by the PCRRLM. PCRRLM, pathologic complete response ratio of liver metastases.

Compared to patients with a PCRRLM ≥0.50, those with a PCRRLM <0.50 were more likely to experience intrahepatic recurrence rather than extrahepatic recurrence (intrahepatic recurrence, *p* = 0.045; extrahepatic recurrence, *p* = 0.941; Figures 2A,B). In addition, patients with PCRRLM <0.50 were more likely to suffer early recurrence (within 6 months from liver resection) than those with a PCRRLM ≥0.50 (*p* = 0.015, Figure 2C).

The results of univariate and multivariate Cox analyses predicting OS and RFS are shown in Tables 4, 5. Several clinicopathological factors significantly associated with worse OS were identified, including a PCRRLM <0.50, multiple metastases number (≥3), positive lymph node metastases, metachronous CRLM, larger liver lesion size (≥3 cm), double lobe involvement, CRS ≥3 points, and TBS ≥5 points. In the multivariate analysis, synchronous liver metastases (vs. metachronous, HR [95% CI]: 0.59 [0.41–0.85], *p* = 0.005) and TBS points (≥5 vs. < 5, HR [95% CI]: 1.58 [1.02–2.45], *p* = 0.042) were independent predictive factors for OS. The following variables included in the Cox regression, analyzed by backward stepwise selection using the AIC, were associated with RFS: PCRRLM, positive lymph node metastases, chemotherapy regimen, number of chemotherapy cycles, CRLM number, CRLM diameter, double lobe involvement, TBS ≥5 points, and CRS ≥3 points. However, in the multivariable analysis, only PCRRLM (≥0.50 vs. < 0.50, HR [95% CI]: 0.67 [0.46–0.99], *p* = 0.043), lymph node status (positive vs. negative, HR [95% CI]: 1.46 [1.04–2.05], *p* = 0.028) and TBS (≥5 vs. < 5, HR [95% CI]: 1.44 [1.02–2.04], *p* = 0.038) were independently associated with RFS.

**TABLE 4:** Univariable and multivariable Cox regression analyses of the predictors of overall survival.

Variables	Univariable		Multivariable	
	HR (95% CI)	*p* value	HR (95% CI)	*p* value
Age (years): ≥55 vs. < 55	0.93 (0.68–1.27)	0.644		
Sex: male vs. female	1.12 (0.79–1.57)	0.532		
PCRRLM: ≥ 0.50 vs. < 0.50	0.61 (0.38–0.99)	**0.045**	0.75 (0.46, 1.23)	0.256
Primary site: colon vs. rectum	0.88 (0.64–1.21)	0.427		
Lymph node status: positive vs. negative	1.61 (1.17–2.21)	**0.003**	1.47 (0.97, 2.24)	0.070
CEA (ng/ml): >5 vs. ≤ 5	1.28 (0.93–1.77)	0.135		
Chemotherapy regimen: oxaliplatin-based vs. irinotecan-based	0.69 (0.50–0.95)	0.076		
Chemotherapy cycles: > 4 vs. ≤ 4	0.88 (0.65–1.21)	0.441		
Synchronous LM: yes vs. no	0.60 (0.42–0.86)	**0.005**	0.59 (0.41, 0.85)	**0.005**
LM number: ≥ 3 vs. < 3	1.56 (1.14–2.15)	**0.005**	1.18 (0.76, 1.83)	0.462
LM diameter (cm): ≥ 3 vs. < 3	1.37 (1.00–1.87)	**0.048**	1.01 (0.69, 1.50)	0.944
Hepatic lobe involvement: double vs. single	1.54 (1.13–2.12)	**0.006**	1.11 (0.75, 1.64)	0.617
CRS: ≥ 3 vs. < 3	1.84 (1.35–2.52)	**<0.001**	1.29 (0.84, 1.98)	0.246
TBS: ≥ 5 vs. < 5	1.88 (1.37–2.58)	**<0.001**	1.58 (1.02, 2.45)	**0.042**
Postoperative chemotherapy: yes vs. no	0.72 (0.51–1.03)	0.068		

Abbreviations: CEA, carcinoembryonic antigen; CI, confidence interval; CRS, clinical risk score; LM, liver metastases; HR, hazard ratio; PCRRLM, pathologic complete response ratio of liver metastases; TBS, tumor burden score. The bold values in table indicate *p* < 0.05.

**TABLE 5:** Univariable and multivariable Cox regression analyses of the predictors of recurrence-free survival.

Variables	Univariable		Multivariable	
	HR (95% CI)	*p* value	HR (95% CI)	*p* value
Age (years): ≥55 vs. < 55	0.90 (0.70–1.16)	0.407		
Sex: male vs. female	1.16 (0.88–1.53)	0.300		
PCRRLM: ≥ 0.50 vs. < 0.50	0.63 (0.43–0.92)	**0.016**	0.67 (0.46, 0.99)	**0.043**
Primary site: colon vs. rectum	1.17 (0.90–1.51)	0.238		
Lymph node status: positive vs. negative	1.38 (1.07–1.79)	**0.012**	1.46 (1.04, 2.05)	**0.028**
CEA (ng/ml): > 5 vs. ≤ 5	1.29 (0.99–1.68)	0.055		
Chemotherapy regimen: oxaliplatin-based vs. irinotecan-based	0.64 (0.49–0.83)	**0.004**	0.78 (0.59, 1.02)	0.073
Chemotherapy cycles: > 4 vs. ≤ 4	0.65 (0.50–0.84)	**0.001**	0.80 (0.61, 1.05)	0.102
Synchronous LM: yes vs. no	0.84 (0.61–1.15)	0.267		
LM number: ≥ 3 vs. < 3	1.69 (1.31–2.18)	**<0.001**	1.42 (0.99, 2.05)	0.059
LM diameter (cm): ≥ 3 vs. < 3	1.34 (1.04–1.72)	**0.022**	1.10 (0.81, 1.49)	0.550
Hepatic lobe involvement: double vs. single	1.50 (1.16–1.94)	**0.002**	1.03 (0.75, 1.41)	0.864
CRS: ≥ 3 vs. < 3	1.56 (1.21–2.01)	**0.001**	1.03 (0.73, 1.44)	0.884
TBS: ≥ 5 vs. < 5	1.93 (1.49–2.49)	**<0.001**	1.44 (1.02, 2.04)	**0.038**
Postoperative chemotherapy: yes vs. no	1.03 (0.76–1.39)	0.846		

Abbreviations: CEA, carcinoembryonic antigen; CI, confidence interval; CRS, clinical risk score; LM, liver metastases; HR, hazard ratio; PCRRLM, pathologic complete response ratio of liver metastases; TBS, tumor burden score. The bold values in table indicate *p* < 0.05.

## Discussion

In this study, we retrospectively analyzed the accumulated cases of CRLM in our hospital in an attempt to identify potential prognostic indicators for these patients. We proposed the PCRRLM, a new and potentially better measurement of pathologic response in patients with CRLM after preoperative chemotherapy and resection, and assessed its value for predicting prognosis and recurrence. Detailed analysis confirmed that patients with a PCRRLM ≥0.50 had a highly favorable prognosis and a significantly lower incidence of recurrence, suggesting that PCRRLM could be a feasible clinicopathological indicator.

Although the strength of the pathologic response after preoperative chemotherapy has generally been accepted as a prognostic factor over the years, the optimal grading system to evaluate this parameter is still a matter of debate. A conventional assessment was semi-quantitatively estimating the proportion of residual cancer cells in relation to the total tumor area, and the mean of the values was used when there was more than one metastasis [21]. Some other researches only considered patients who had achieved full PCR in all tumors [23, 28]. However, in clinical practice, most patients had multiple liver metastases, among which only a small percentage achieved full PCR in all tumors. Practically, the number of liver metastases was the most frequently included prognostic factor in all studies of CRLM [34]. Moreover, achieving PCR in all metastases proved not to be necessary, while at least one tumor showed PCR was also beneficial to prognosis [35]. The conventional method did not consider the number of metastases, and the applicability was limited due to the low odds of PCR in all tumors. In contrast, PCRRLM carefully taken into account the number and heterogeneity among multiple metastases, allowing for better characterization of pathologic response for patients with multiple CRLMs.

Another popular index for pathologic response was tumor regression grade (TRG), a 5-point scoring system firstly proposed for esophageal tumor and then introduced into the field of colorectal cancer by Dworak *et al.* [36] TRG is semi-quantitatively determined by the amount of residual tumor cells versus the amount of fibrosis, with a TRG of 4 indicating no evidence of any treatment effect and a TRG of 0 indicating a complete response with no viable tumor remaining. The interpretation of TRG in patients with multiple liver metastases is flawed because these patients are mostly categorized according to the worst grade (namely, the highest TRG), which may not reflect the heterogeneity observed among metastatic lesions. As has been documented in many studies[8, 12–14], the number of metastases has a significant impact on patient outcome. TRG essentially ignores the regression status of all but the worst tumor. By comparison, our methods take into account the regression status of all resected tumors from a holistic point of view. Moreover, because TRG is a semiquantitative measurement, it is inevitably influenced by the subjectivity of pathologists. The prognostic value of TRG can hardly be observed across each tier (from TRG 4 to 0); hence, a variety of complex grouping strategies have been applied by researchers, all of which have lacked rigor [37–39] (e.g., TRG 4 *v* 2+3 *v* 0+1, TRG 4+3 *v* 2 *v* 0+1, or TRG 4+3 *v* 2+1+0). In contrast, our methods avoid this subjectivity and grouping complexity to some extent and proved to be an excellent predictor for OS and RFS. Thus, the PCRRLM has the potential to be a better portrayal of pathologic response after chemotherapy.

One of the inherent drawbacks of applying the PCRRLM in clinical practice is the difficulty of assessing it in a noninvasive manner prior to surgery. Therefore, it would be considerably valuable to preoperatively identify patients who are likely to achieve a favorable pathologic response. In this study, we determined that age <55 years old and preoperative CEA ≤5 ng/ml are strong predictors of PCRRLM ≥0.50. In addition, a primary tumor site in the rectum, CRLM diameter of <3 cm, and TBS <5 points were all identified as predictors in the univariate analysis. The correlations among small tumor size, low CEA levels, and PCR have been well confirmed by previous studies [21, 23, 28, 40, 41]. Some researchers [23, 28] used a significantly elevated CEA level (e.g., 20 or 30 ng/ml) as the classification standard, while in our study, the critical value of a normal concentration (5 ng/ml) was applied. Our results further demonstrated the value of a normal preoperative CEA level and small CRLM diameter in predicting a favorable pathologic response, since the probability was 34.2% (25/73) when two factors were present and 6.3% (7/111) when only one was present. Another factor that has been found to be related to the PCRRLM is the primary tumor location. Several studies [42–45] have reported a relatively better response to chemotherapy and favorable survival in patients with metastases from left-side primary tumors. We did not observe a significant difference between patients with left- and right-sided primary tumors, but we found that patients with a primary tumor site in the rectum, which can be classified as left-sided colon in histogenesis [46], were more likely to have PCRRLM ≥0.50. This may be due to the genetic heterogeneity of primary tumors at different sites, as they differ in terms of embryologic development, blood supply, macroscopic pathology, and clinicopathologic parameters [47]. Moreover, we identified a nonnegligible correlation between a low TBS and better PCRRLM. TBS was first proposed by Sasaki et al. [31] by combining the size and number of CRLMs in a continuous rather than dichotomous stratification and was proven to have a higher prognostic value than the traditional CRS system. Ruzzenente et al. [48] found that a reduction in the TBS indicated a response to preoperative chemotherapy and could serve as a predictor of survival. Sasaki et al. [49] further validated that imaging-based TBS had the same strength as pathology-based TBS in predicting OS preoperatively. Considering the potential of a low TBS and high PCRRLM, our results further demonstrate the significant correlation between the TBS and pathologic response and provide evidence of a method to predict pathologic response by calculating the TBS using preoperative radiologic cross-sectional imaging, which may make it unnecessary to wait for the results from resected tumors after surgery.

The long-term prognosis was much better for patients who had a higher PCRRLM than for those who did not. In our study, the proportion of patients who achieved pathologic complete response (PCR) was 4.9% (15/305), which is similar to previous studies [21, 23, 28]. Owing to this low incidence, the application of PCR in clinical practice is limited, and few changes in patients may bias the results. Considering that PCR can improve the prognosis of patients with only one metastatic liver lesion [35], we relaxed the inclusion criteria and categorized patients into two groups: the high-PCRRLM group (≥0.50) and the low-PCRRLM group (<0.50). The new criteria categorized 14.4% (44/305) of patients into the high group and 85.6% (261/305) into the low group. After validation, a higher PCRRLM was found to serve as a good predictor of favorable survival, with a remarkable 5-year OS rate of 62.75% in the high group and 41.06% in the low group (*p* = 0.045). For recurrence-free survival, the 5-year RFS rate in the high-risk group was 27.18%, whereas that in the low-risk group reached only 16.82% (*p* = 0.016). The excellent prognosis of the high-PCRRLM group, especially in terms of recurrence-free survival, can be explained by their favorable response to preoperative chemotherapy.

The response to chemotherapy was limited among patients with a PCRRLM less than 0.50, and their prognosis was relatively poor. Hence, for these patients, the follow-up interval should be shortened, as closer clinical monitoring is needed. A low PCRRLM also suggests that the chemotherapy regimen should be modified. Some studies have reported a relatively higher tumor response after oxaliplatin-based preoperative chemotherapy than irinotecan-based preoperative chemotherapy [21, 28, 39, 50], although this correlation was not found in our research. A study conducted in a Chinese cohort [40] also reported a better pathologic response when cetuximab was combined with irinotecan-based chemotherapy. Therefore, a timely assessment of the PCRRLM after hepatectomy can provide clinicians with guidance and a reference for selecting postoperative chemotherapy, contributing to a better therapeutic effect.

This study had some limitations, which should be acknowledged. First, this was a retrospective analysis that included only a limited number of patients from a single institution. Incorporating the grouped chemotherapy and targeted therapy regimens into the analyses would make it difficult to obtain good reproducible results, as this was a small cohort study. Therefore, the findings need further validation in a larger prospective cohort of patients. Second, the mutation status of some genes, such as *RAS* and *BRAF*, have been confirmed as significant prognostic factors and widely applied in clinical practice [51, 52], but we did not include them in the analysis because these data were unavailable for the majority of patients in this study. We also did not incorporate the TRG index in this study. For a large number of liver metastases, there are many possibilities of TRG, and there is no clear standard for which TRG should be selected as the final TRG. It is inappropriate to use single-tumor TRG measurements for tumor regression, so a comparison between PCRRLM and TRG is not done. Moreover, PCRRLM was not available for patients who had undergone radiofrequency ablation. Prospective randomized trials are needed in the future to further verify the reliability and validity of the PCRRLM.

In conclusion, we proposed the PCRRLM as a novel grading system for the pathologic response of patients after the resection of CRLM treated with preoperative chemotherapy. The long-term RFS of patients who achieved a PCRRLM ≥0.50 was extremely favorable, and most of them showed a significantly lower tendency toward recurrence. The PCRRLM may provide an objective and precise evaluation of the pathologic response of patients with CRLM.

## Data Availability

The datasets presented in this study can be found in online repositories. The names of the repository/repositories and accession number(s) can be found below: www.researchdata.org.cn, with the approval number RDDA2022115638.
